# Leveraging Pretrained Neural Network Models for the Classification of Tumor Cells Analyzed by Label-Free Phase Holotomographic Microscopy

**DOI:** 10.34133/csbj.0111

**Published:** 2026-06-09

**Authors:** Leonor V. C. Losa, Temple A. Douglas, Lia Santos, Raquel Monteiro, Isabel Calejo, Raphaël F. Canadas, Jana B. Nieder

**Affiliations:** ^1^ INL—International Iberian Nanotechnology Laboratory, Nieder Group on Quantum-,Bio- and Nanophotonics, 4719-330 Braga, Portugal.; ^2^ Department of Biomedicine, Faculty of Medicine, University of Porto, 4200-450 Porto, Portugal.; ^3^ RISE-Health, Faculty of Medicine, University of Porto, 4200-450 Porto, Portugal.

## Abstract

We present an innovative methodology for label-free, high-resolution imaging using phase holotomographic microscopy, coupled with neural network models for the classification of cancer cells. Using 3-dimensional phase holotomographic microscopy, we imaged live A549 lung cancer cells with and without paclitaxel, converted stacks to 2-dimensional maximum-intensity projections, and evaluated pretrained convolutional networks (VGG16, ResNet18, DenseNet121, and EfficientNet-B0) for binary classification of treatment status. EfficientNet-B0 achieved 96.9% accuracy on unsegmented images. Refractive index analysis revealed bimodal distribution in treated cells, reflecting heterogeneous biophysical responses to paclitaxel exposure and supporting the network’s ability to detect subtle, label-free indicators of drug action. As further proof of concept, the same pipeline separated holotomographic images of label-free, high- versus low-grade urothelial cancer cells with high accuracy (90.6%). These findings highlight the potential of integrating label-free holotomographic imaging with deep learning techniques for rapid and efficient classification of tumor cells, paving the way for advancements in treatment optimization and personalized diagnostic strategies.

## Graphical Abstract

**Figure:**
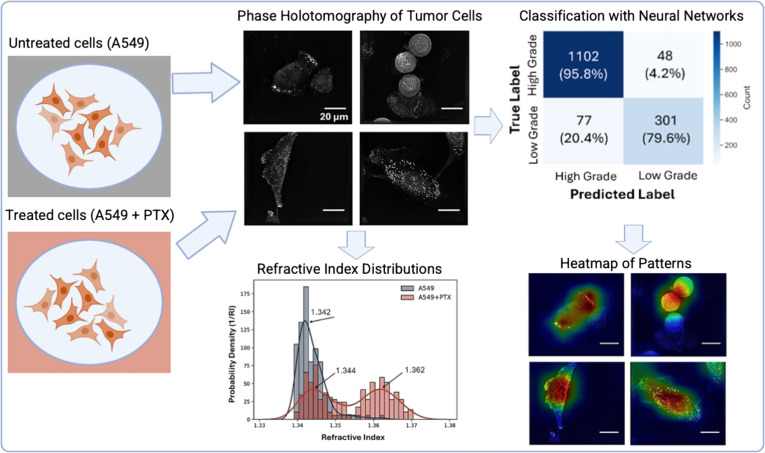

## Introduction

At present, cancer is among the leading causes of death worldwide, accounting for approximately 9.74 million deaths, and its incidence is expected to increase to an estimated 16.9 million deaths by 2045 [1]. Among the various types of cancer, lung cancer is forecasted to be the leading cause of cancer-related deaths in the world. By 2045, 4.25 million new cases of lung cancer and 3.24 million deaths are expected annually [2]. In this context, understanding the biophysical properties of tumors at a deeper level is essential, particularly for identifying factors that can improve diagnostics and therapeutic outcomes, or for optimizing more efficient protocols for diagnostics and treatment [3–5].

A major challenge in treating this type of cancer is its heterogeneity in both its treatment response and clinical characteristics, even among tumors of the same pathological type [6–8]. This variability makes it challenging to accurately predict an individual’s treatment response.

To better address the complexity introduced by tumor heterogeneity and improve prediction accuracy despite this variability, computational approaches, particularly machine learning (ML), have been increasingly applied to cancer research. These algorithms can analyze large datasets of medical images and extract patterns indicative of cellular response to an anticancer treatment, for example [9,10]. Convolutional neural networks (CNNs), including transfer learning with pretrained models, have shown strong results in predicting therapy response [11,12]. For instance, Change *et al.* [13] used VGG16 to predict chemotherapy response in non-small cell lung cancer computed tomography (CT) scans with an accuracy of 88.3% with an AUC (area under the receiver operating curve) of 0.982, representing a high model fit to the data.

Even though these approaches have proven successful, these types of images do not capture cellular-level responses to chemotherapy. This step has been predicted to be an important future methodology for optimizing cancer treatment [14]. Thus, there has been increased interest in live-cell imaging techniques.

Since living cells are naturally transparent, it is challenging to image them with sufficient contrast to observe and analyze their structures. This limitation has been mitigated by using fluorescent dyes, which enhance visibility but introduce effects such as interference with biological processes, phototoxicity, and photobleaching [15,16].

As an alternative, phase holotomography (PHT) leverages the unique refractive index (RI) properties of cellular components as an intrinsic imaging contrast [17]. This technique enables the visualization of live subcellular features, cellular density, and morphology without the need for dyes and fluorescent labels [18,19]. Recent work has shown that coupling ML-type techniques with the high resolution of holotomographic microscopy can computationally simulate hematoxylin and eosin staining in pathology samples, potentially automating and speeding up pathology workflows and indicating the potential for ML–holotomography hybrid approaches to pathology analysis [20].

The method relies on a partially coherent laser excitation source that is split into a sample and reference beam via a Mach-Zehnder-type interferometric configuration, from which quantitative phase information is reconstructed into a 3-dimensional (3D) RI map [21].

PHT employs low-energy light that minimally perturbs the specimen, reducing phototoxic effects, making it ideal for imaging living cells [22]. Additionally, PHT’s numerical refocusing capability enables detailed imaging of cells in 3D volumes [23].

Building on the capability of PHT systems to provide detailed, label-free insights into cellular responses to therapeutic interventions, this study leverages the combined capabilities of phase holotomographic imaging and ML pretrained CNN models to objectively assess differences between A549 lung cancer cells treated and untreated with paclitaxel (PTX).

This study represents the first known demonstration of PHT coupled with ML to differentiate chemotherapy-treated cells from healthy cells. This methodology provides a first proof of concept that advanced holotomography and ML techniques could be used in the future to evaluate treatment efficacy in live-cell therapy optimization systems.

## Materials and Methods

### Experimental design

The objective of this study was to determine whether label-free PHT images of lung cancer cells could be used in combination with deep learning to classify PTX-treated versus untreated cells. We designed experiments in 3 stages: (a) culture and drug treatment of A549 cells, (b) image acquisition with the PHM system, and (c) data preprocessing and classification using a pretrained CNN model. To assess pipeline generalizability, we also tested the optimized pipeline on urothelial cancer cells.

### Cell line and culture

Human lung adenocarcinoma A549 cells (ATCC, Virginia, USA) were cultured in Dulbecco’s Modified Eagle Medium supplemented with 10% fetal bovine serum (FBS) and 1% penicillin–streptomycin (PS). Cells were maintained at 37 °C in a humidified incubator with 5% CO₂ and passaged at ~70% confluence.

Furthermore, we used commercially available urothelial cancer cell lines from high-grade and low-grade patient diagnostics. Cells were cultured in Roswell Park Memorial Institute (PAN-Biotech, Germany) 1640 culture medium supplemented with 10% FBS and 1% PS.

Tumor-derived cells were obtained from an ongoing clinical study conducted in collaboration with São João Hospital. All procedures were performed in accordance with the ethical standards of the institutional research committee and the Declaration of Helsinki; the appropriate review board granted ethical approval.

Following surgical excision, tumor tissue samples were processed under sterile conditions to remove residual vascular material and obtain clean epithelial tumor fragments. The tissue was mechanically dissociated into small explants and placed in standard cell culture plates. Growth medium was added, and explants were incubated at 37 °C in a humidified 5% CO₂ atmosphere. Cell outgrowth and migration from the explants were monitored by phase-contrast microscopy.

Culture supernatant was collected periodically and used in subsequent cell culture steps. Cells were expanded through serial subculturing until a stable adherent cell population displaying consistent proliferation and morphology was established.

Urothelial cancer cells were fixed in suspension with 4% paraformaldehyde for 30 min as a preprocessing step for phase holotomographic image acquisition.

### Calculation of PTX IC_50_

To determine the half-maximal inhibitory concentration (IC_50_) of PTX, 10^4^ A549 cells/well were seeded in 96-well plates and treated with PTX concentrations ranging from 0.0005 to 5 μM. After 24 h of exposure, cells were washed with phosphate-buffered saline and incubated with resazurin (10 μg/ml, Sigma-Aldrich) for 4 h at 37 °C. Fluorescence intensity was measured by exciting at 560 nm and detecting at 590 nm, using a microtiter plate reader (Biotek Synergy H1, Agilent, USA). An IC_50_ concentration of 0.02738 μM was calculated using an [Inhibitor] *vs.* response − variable slope (4-parameter) model, found in the Supplementary Materials.

### Chemotherapy drug treatment

For imaging, A549 cells were seeded in culture dish (81218-200, Ibidi, Germany) chambers and allowed to adhere for 24 h at 37 °C with 5% ${\mathrm{CO}}_{2}$. After this period, the culture medium was replaced with fresh phenol red-free medium containing 0.02849 μM PTX, corresponding to the ${\mathrm{IC}}_{50}$ for this cell line. After 24 h in the medium with PTX, we imaged the cells.

### Phase holotomographic image acquisition

Three-dimensional phase holotomographic images were acquired using a phase holotomographic microscope (3D Cell Explorer, Nanolive S.A., Switzerland) that splits a semi-coherent laser (520 nm) into a Mach-Zehnder configuration, with one arm passing through the sample and the other serving as a reference for reconstructing a digital hologram.

The microscope utilized a dry 60×/numerical aperture 0.8 objective and a complementary metal–oxide–semiconductor camera (IMX174 CMOS, Sony, Korea). The system operates with a class 1 laser (*λ* = 520 nm, 0.2 mW/$mm^{2}$).

Interference between the reference and sample beams generated an interference pattern, which was then transformed by the STEVE software (Nanolive S.A., Switzerland) into 96 2-dimensional (2D) holograms, subsequently reconstructed into 3D tomograms.

Environmental controls during imaging were rigorously maintained. Two hours before each experiment, the gas mixer was set to 0.5 l/min for air and 0.02 l/min for CO_2_, and the temperature controller was set to 37 °C. An air pump was activated, and the 2 chambers within the incubator were filled with distilled water to ensure high humidity and prevent sample evaporation. The maximum field of view for image acquisition was 90 × 90 × 30 μ$m^{3}$.

### Data preprocessing

To prepare volumetric data for analysis, we first trimmed noninformative slices from the top and bottom of each 3D image stack, removing sections that contained only background signal or reconstruction noise and no visible cellular content. This ensured that the subsequent projections represented only the biologically relevant regions of the sample. After trimming, maximum-intensity projection (MIP) images were generated to obtain 2D representations of the 3D datasets. Each original stack consisted of 96 slices, and each projected image was 512 × 512 pixels, corresponding to an imaging area of approximately 90 × 90 μm.

For the ML pipeline, all images were resized to 224 × 224 pixels to match the input size required by the pretrained CNN architectures used in this study.

### Image segmentation

For CNN classification of the urothelial cancer cell lines and RI calculation of all cell lines, we used *Cellpose* to segment multicell images into individual cells. The segmentation process produced binary masks, where each detected cell was assigned a unique label corresponding to its outline. These masks were used to extract individual cells from the MIP images, generating cropped single-cell images. Because the segmented cells varied in size and shape, each cropped image was centered on a black background and padded with black pixels to create a uniform 224 × 224 pixel frames suitable for input into the ML models.

Segmentation performance varied according to cell type. For A549 cells imaged live, segmentation was limited by frequent cell overlap and boundary ambiguity, resulting in the exclusion of some images from the segmented dataset. For this reason, segmented images were used only for RI analysis, not for CNN classification. In contrast, urothelial cancer cells were chemically fixed before imaging, resulting in clearer boundaries and more stable morphology, which enabled successful segmentation of all imaged cells.

### Deep learning pipeline

We adapted 4 pretrained ImageNet CNNs (EfficientNet-B0, DenseNet121, ResNet18, and VGG16) to grayscale PHT images. Since our images are single-channel, the original 3-channel input convolutions were replaced by single-channel versions initialized from a normal distribution (*μ* = 0, *σ* = 0.001). All other layers retained pretrained weights. All models were originally trained on ImageNet (1,000 classes, RGB input), so their final fully connected classifier layers were replaced with 2-unit output layers corresponding to the 2 study classes (untreated and PTX-treated A549 cells, and high grade *vs.* low grade).

For EfficientNet-B0, the output of the average pooling layer (1,280 features) was passed to a fully connected layer of 640 units, followed by dropout. A second dropout was added before the final classification layer (2 output units).

For DenseNet121, we inserted a dropout layer between the 1,024-dimensional pooled features and the final classifier, and changed it to 2 output units.

For VGG16, the 2 existing dropout layers were retained, but their probabilities were optimized rather than fixed at 0.5, and the output layer was replaced with a 2-unit classifier.

For ResNet18, we added a dropout layer between the 512-dimensional pooled features and the output layer (2 units).

All dropout probabilities were tuned using *Optuna’s Bayesian* optimization (TPESampler) within the range [0.25 to 0.48] for the first dropout and [0.18 to 0.55] for the second. *Optuna* also optimized the learning rate ([0.0002 to 0.0015]) and weight decay ([0.0005 to 0.05]), targeting the Matthews correlation coefficient (MCC) as the objective metric.

Training used 5-fold stratified cross-validation with early stopping (patience = 15) and data augmentation (random flips and rotations) applied only to training subsets. Images were resized to 224 × 224 pixels, and *z*-score normalized using training statistics per fold to prevent data leakage. To ensure robustness against initialization variance, all experiments were repeated 3 times with different random seeds (53, 65, and 88), and metrics were averaged across the 3 runs.

Performance was reported for both the single best model (highest validation accuracy) and an ensemble approach. In the ensemble, multiple independently trained models contributed predictions for each test sample, and the averaged *softmax* probabilities were used to determine the final class output. This strategy reduces the impact of variance from random initialization and training splits, improving overall prediction stability.

## Results

### PHT imaging of A549 cells

**Fig. 1.**
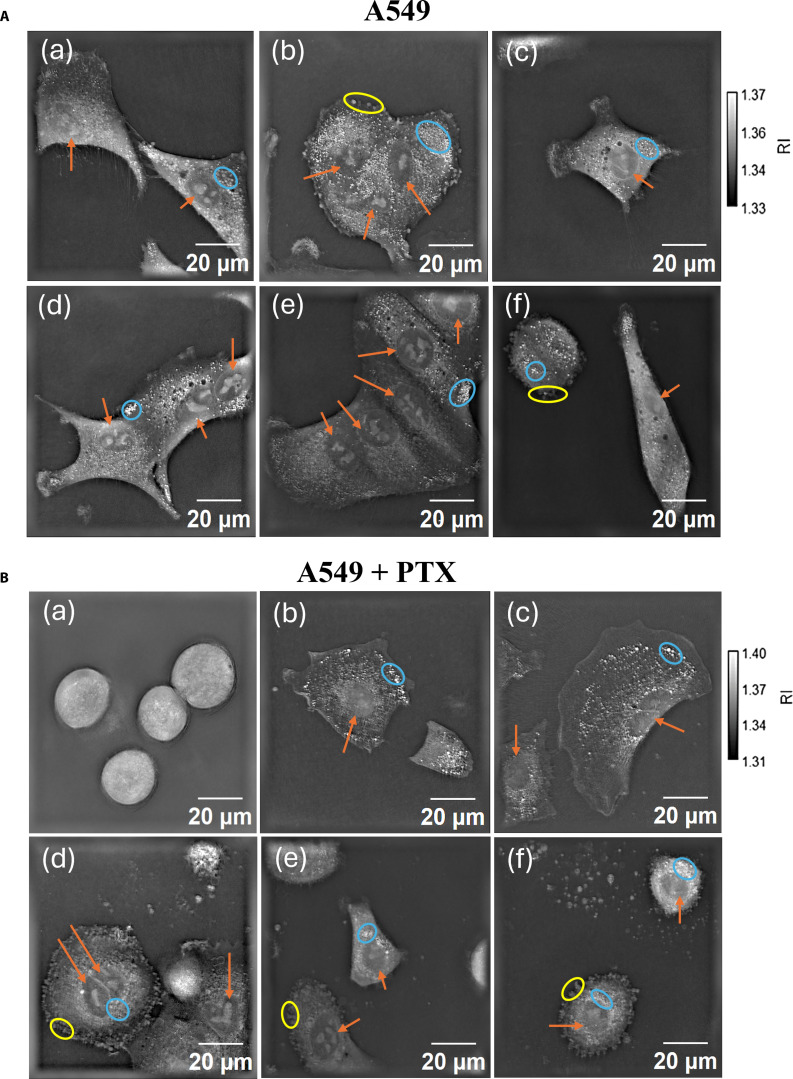
Selected phase holotomographic tomography (PHT) images of untreated and treated cancer cells. (A) Representative images of untreated A549 cells (a to f). (B) Representative image of treated A549 + PTX (a to f). The nucleus is highlighted by orange arrows. Blebs and vesicles are highlighted by yellow and blue ellipses, respectively.

Label-free PHT revealed distinct morphological differences between untreated (Fig. 1A) and PTX-treated (Fig. 1B) A549 cells. Untreated cells displayed heterogeneous adherent morphologies, with well-defined nuclei and vesicles. In contrast, PTX-treated cells displayed a variety of morphologies, including many rounded, detached cells exhibiting typical apoptotic-like features, such as membrane blebbing. These morphological changes may result from PTX’s known mechanism of action in inducing cell death or from other known resistance pathways [24,25].

**Fig. 2.**
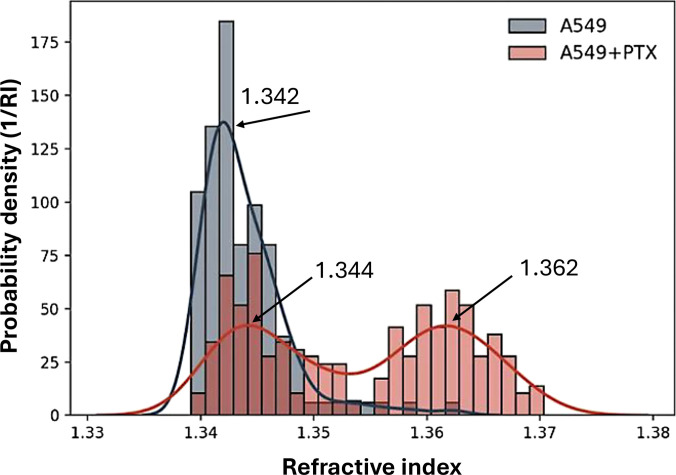
Refractive index distribution for A549 cells (gray) and A549 + PTX cells (red), using only segmented cells to avoid averaging with background.

Quantitative analysis of the RI distributions calculated for cells segmented using the *Cellpose* pipeline to remove background from RI averaging demonstrated that PTX treatment caused a measurable increase in cellular density [26,27]. The mean RI increased from 1.3438 in untreated A549 cells to 1.3539 in treated cells. Each data point in the RI distribution corresponds to the average RI of a single segmented cell, not individual pixels, ensuring that variations reflect cell-level biophysical differences. As shown in Fig. 2, the RI distribution also transitioned from unimodal (in untreated A549 cells) to bimodal (in PTX-treated cells). This shift likely arises from the use of the 24-h IC_50_ concentration of PTX, which is cytotoxic, generating a mixed population of viable and apoptotic cells at 24 h. It could also be due to changes in cellular composition resulting from PTX’s mechanism of cell death, tubulin stabilization, and cellular arrest in G2 or M phase. The lower RI mode likely corresponds to cells that remain viable and morphologically similar to untreated cells. In contrast, the higher RI mode may reflect cells undergoing apoptosis or other forms of cell death. Statistical testing was performed on this dataset. The Kolmogorov–Smirnov test gave values *D* = 0.422, *P* = 1.465e−25 (significant). The Mann–Whitney test gave a *U* value of *U* = 25372.5, *P* = 3.058e−26 (significant).

### Classification of unsegmented A549 images

We trained 4 pretrained CNNs (VGG16, ResNet18, DenseNet121, and EfficientNet-B0) on maximum-intensity projections of unsegmented PHT images, comprising 359 untreated A549 images and 282 PTX-treated A549 images [28–31]. Model evaluation was performed using 3 independent 5-fold cross-validation runs to ensure statistical robustness and mitigate data-split bias [32].

Across all architectures, EfficientNet-B0 consistently outperformed the other models, achieving 96.9% ± 0.6% accuracy, 96.9% ± 0.6% F1-score, and MCC = 0.94 ± 0.01 (Table 1). In comparison, VGG16 and ResNet18 achieved slightly lower MCC values of 0.89 and 0.91, respectively, whereas DenseNet121 approached EfficientNet-B0 with an MCC of 0.93.

**Table 1.** Test-set results for selected metrics across 3 runs for different pretrained models applied to the PTX- treated and untreated A549 datasets. Bracketed values represent 95% confidence intervals.

Model	Accuracy ± SD (%)	F1-score ± SD (%)	MCC ± SD
VGG16	94.57 ± 2.19 [92.8–96.3]	94.59 ± 2.18 [92.8–96.3]	0.893 ± 0.041 [0.869–0.917]
ResNet18	95.35 ± 0.64 [93.7–97.0]	95.35 ± 0.65 [93.7–97.0]	0.906 ± 0.014 [0.883–0.929]
DenseNet121	96.38 ± 0.37 [94.9–97.8]	96.39 ± 0.37 [94.9–97.8 ]	0.927 ± 0.08 [0.907–0.947]
EfficientNet-B0	96.90 ± 0.63 [95.6–98.2]	96.89 ± 0.64 [95.5–98.2]	0.938 ± 0.013 [0.919–0.957]

**Fig. 3.**
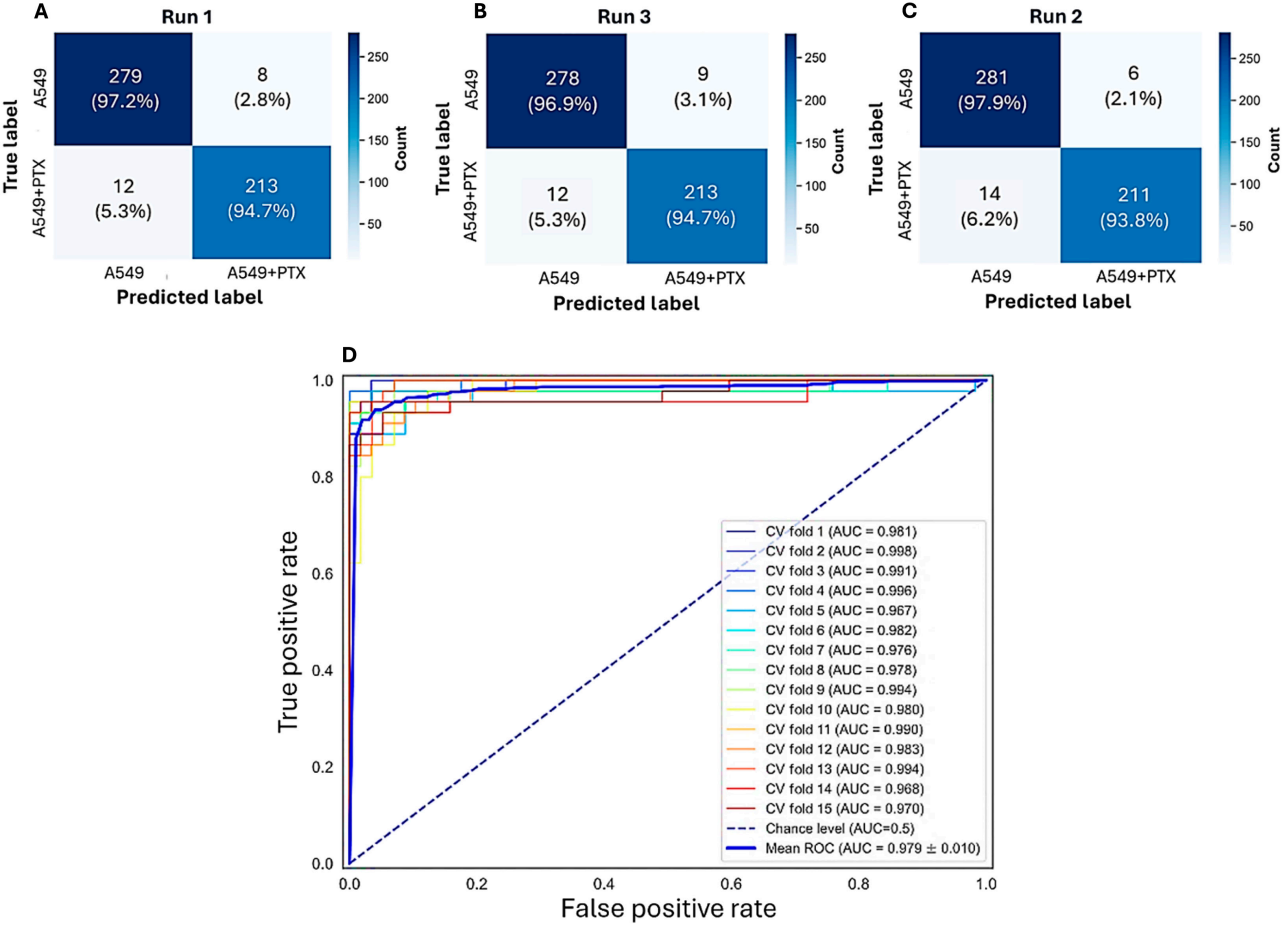
Cross-validation confusion matrix for the EfficientNet-B0 model on A549 cells. (A) Run 1, (B) run 2, and (C) run 3. (D) Receiver operating characteristic (ROC) curve of the EfficientNet-B0 model for the cross-validation (CV) across the 3 runs, for the unsegmented dataset. Folds 1 to 5 are from run 1, folds 6 to 10 are from run 2, and folds 11 to 15 are from run 3.

Because EfficientNet-B0 demonstrated superior performance and stability, we selected it for a more detailed analysis. Figure 3 summarizes the validation-stage results obtained during cross-validation for this model only. Figure 3A to C display confusion matrices for the 3 independent runs. Each matrix shows that the model correctly identified nearly all untreated A549 cells as “A549” (true-negative rate >97%) and most PTX-treated cells as “A549+PTX” (true-positive rate >93%). Misclassifications were infrequent (<4%) and occurred in both classes, without a consistent pattern. The precision and recall for the A549 population was 0.96 and 0.97, respectively. The precision and recall for the A549-PTX population was 0.97 and 0.94, respectively. Averaging these recall values provides a balanced accuracy of 96%. Figure 3D presents the receiver operating characteristic (ROC) curves for all cross-validation folds of EfficientNet-B0 (across the 3 runs). Each curve lies close to the top-left corner, indicating excellent sensitivity and specificity. The mean area under the curve (AUC) was 0.98 ± 0.01, confirming the model’s strong discriminative power in distinguishing treated from untreated cells.

**Fig. 4.**
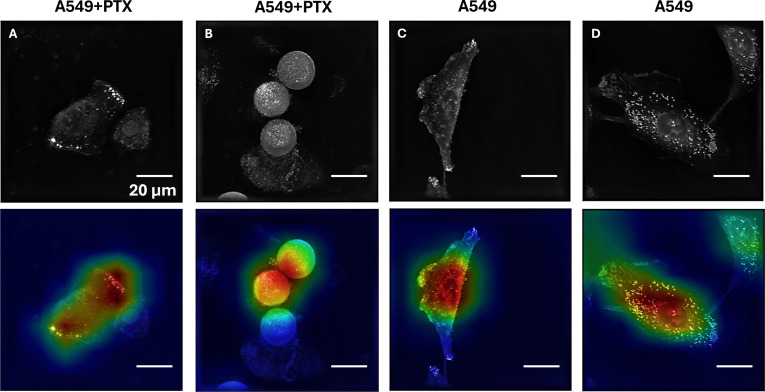
Comparison of selected PHT images (top) and heatmaps (bottom) generated with Grad-CAM++ for the EfficientNet-B0 model on A549 and A549 + PTX datasets. These heatmaps were generated for the correctly predicted (A and B) “A549 + PTX” and (C and D) “A549”. All images are from the test set.

To interpret the model’s internal decision process, we applied Gradient-weighted Class Activation Mapping (Grad-CAM++) [33] to the last convolutional layer of the EfficientNet-B0 model and visualized the resulting attention maps for test-set images (Fig. 4). In these heatmaps, warmer colors (red/yellow) indicate regions contributing most strongly to the model’s prediction. In comparison, cooler colors (blue/green) represent less relevant areas. For PTX-treated A549 cells (Fig. 4A and B), attention was distributed across the cell body, with stronger activations in condensed regions. In contrast, untreated cells (Fig. 4C and D) showed attention concentrated along smoother cellular edges and in internal areas.

The heatmaps showed that the network’s attention was predominantly focused on the cellular regions, particularly within the cell body, rather than on the background. This suggests that model-based decisions are made based on relevant morphological cues rather than artifacts or background noise. Although the highlighted areas did not correspond to specific identifiable subcellular structures, their consistent localization within the cell confirms that EfficientNet-B0 learned to extract meaningful morphological information associated with PTX-induced changes.

Together, these findings demonstrate that EfficientNet-B0 reliably distinguishes between untreated and PTX-treated A549 cells from label-free PHT images.

Additional analyses to evaluate the effect of experimental setup can be found in the Supplemental Materials. Here, we excluded some experiments from initial training and used these batches for testing the model.

### Validation with urothelial cancer cells

Tumor grading is a central pathological measure that reflects how closely tumor cells resemble their normal tissue counterparts, indicating malignancy and clinical aggressiveness. Low-grade tumors are composed of relatively well-differentiated cells, maintaining organized architecture, uniform nuclei, and slower proliferation rates. In contrast, high-grade tumors contain poorly differentiated cells with marked nuclear atypia, irregular shapes, high mitotic activity, and increased invasiveness [34,35].

In urothelial carcinoma, grading plays a decisive role in prognosis and treatment strategy. Low-grade tumors are typically noninvasive and managed conservatively, whereas high-grade tumors are more likely to recur or progress and often require aggressive treatment or surveillance. However, grading currently relies on histopathological staining and visual assessment by expert pathologists, a process that is subjective, labor-intensive, and dependent on the quality of tissue preparation.

The ability to distinguish between tumor grades using label-free, quantitative imaging would offer a valuable alternative for rapid and standardized cancer classification. Because phase holotomographic microscopy measures intrinsic biophysical properties such as cellular RI, density, and morphology, it provides contrast directly related to the underlying state of cell differentiation and malignancy. Integrating this information with deep learning models could therefore enable automated, objective grading of tumor cells across cancer types.

**Fig. 5.**
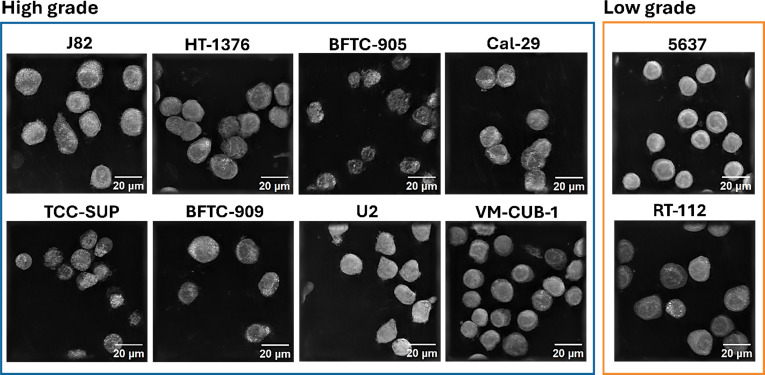
Maximum intensity projection (MIP) phase holotomography (PHT) images of nonadherent bladder cancer cells. Images on the left represent high grade and those on the right denote low grade. The name of the cell line is indicated on top of the image; one image per used cell line is given.

To assess the generalizability of our coupled phase holotomographic microscopy and deep learning pipeline, we tested the EfficientNet-B0 pipeline on a different question, to determine if the model could accurately distinguish between low-grade and high-grade nonadherent, paraformaldehyde-fixed, urothelial cancer cells from 10 different sources (8 bladder cancer cell lines divided into 1,437 cells from 6 high-grade populations and 473 cells from 2 low-grade populations) (Fig. 5). These cell lines display broadly similar rounded morphologies typical of nonadherent fixed cells, as they would be found in urine. Because the variations between them are not visually sufficient for reliable discrimination by inspection alone, this highlights the need for quantitative analysis of intrinsic optical properties and ML-based classification.

**Fig. 6.**
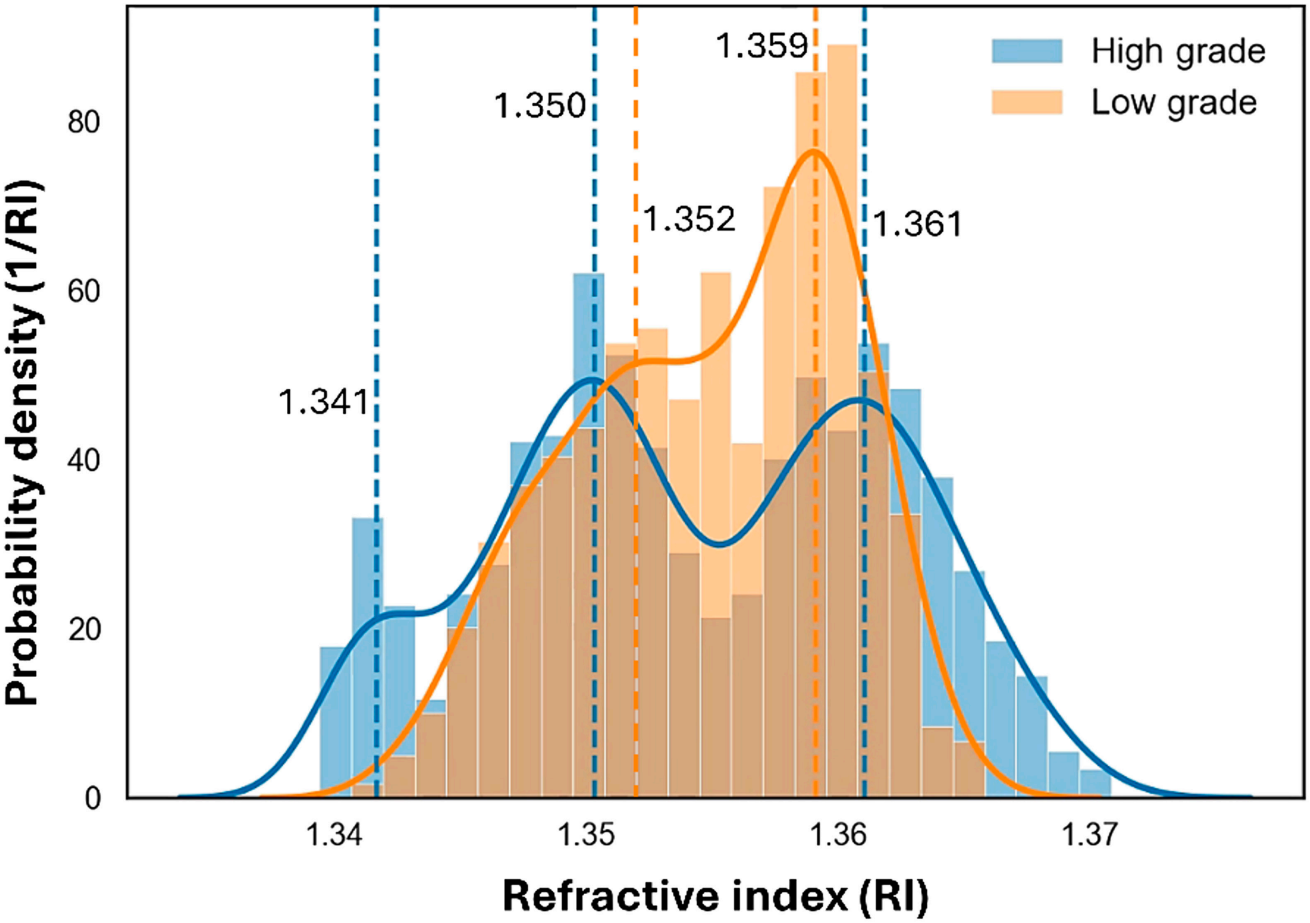
Refractive index distribution for high-grade (blue) and low-grade (orange) bladder cancer cells, using only segmented cells to avoid averaging with background. The refractive index values for the distributions identified by multi-Gaussian fits are indicated.

To investigate whether these subtle differences were associated with measurable biophysical variations, we first analyzed the RI distribution, using only segmented single-cell images. As shown in Fig. 6, low-grade cells exhibited a narrower RI distribution, with peaks at 1.352 and 1.359, while high-grade cells showed broader, multimodal distributions with additional peaks near 1.341, 1.350, and 1.361. These shifts indicate possible increases in internal heterogeneity and density variations in high-grade cells, consistent with their more disorganized morphology. Statistical testing between the populations indicated that these distributions were distinct. The Kolmogorov–Smirnov test gave a result *D* = 0.118, *P* = 9.065e−05 (significant). The Mann–Whitney test result was *U* = 363328.0, *P* = 2.404e−02 (significant).

**Fig. 7.**
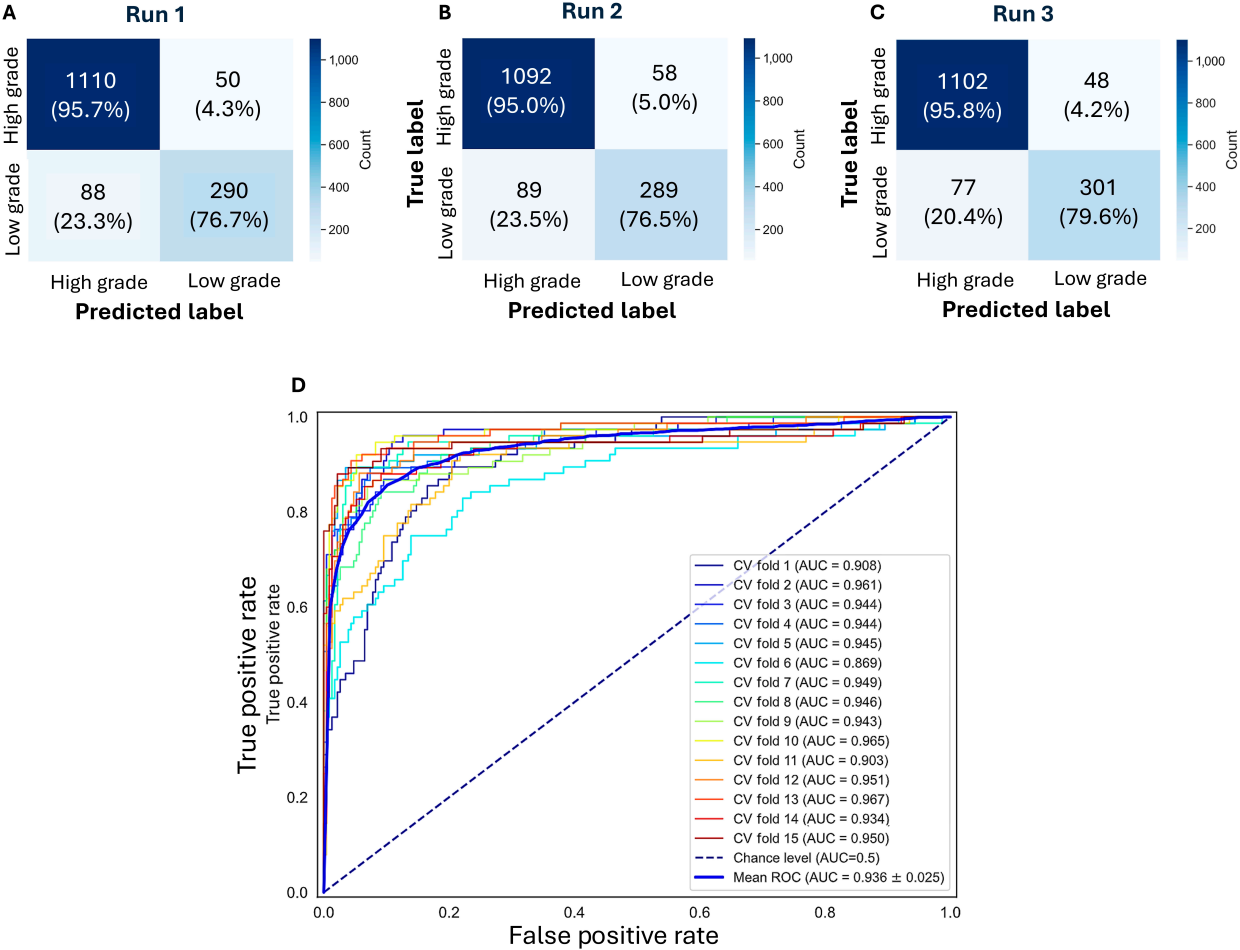
Cross-validation confusion matrix for the EfficientNet-B0 model on bladder cancer cells. (A) Run 1, (B) run 2, and (C) run 3. (D) Receiver operating characteristic (ROC) curve of the EfficientNet-B0 model for the cross-validation (CV) across the 3 runs, for the unsegmented dataset. Folds 1 to 5 are from run 1, folds 6 to 10 are from run 2, and folds 11 to 15 are from run 3.

For this task, the EfficientNet-B0 model was trained on these segmented MIP images using the previously used pipeline and evaluated across 3 independent runs with different random seeds to confirm result stability. Each run used 5-fold cross-validation, and performance was reported on the independent test set. The validation results across the 3 runs are summarized in Fig. 7, where the confusion matrices (A to C) show consistent classification patterns across repetitions. The model reliably distinguished between high- and low-grade cells. The precision and recall for the high-grade population was 0.93 and 0.95, respectively. The precision and recall for the low grade population was 0.85 and 0.78, respectively. Averaging these recall values provides a balanced accuracy of 87%.

The ROC curves for all 15 validation folds (5 folds × 3 runs) are shown in Fig. 7D, demonstrating strong and stable discriminative performance across all runs, with a mean AUC of 0.94 ± 0.03. The narrow AUC variation confirms the model pipeline’s reproducibility and robustness.

Quantitative metrics for both the single best model and the 5-fold ensemble are summarized in Table 2. The ensemble achieved 90.58% ± 0.43% accuracy, 90.24% ± 0.47% F1-score, and an MCC of 0.738 ± 0.013, demonstrating excellent classification performance and high consistency across random initializations.

**Table 2.** Test-set results for selected metrics across 3 runs for the pretrained EfficientNet-B0 model applied to high-grade versus low-grade cancer stage urothelial cancer cells (segmented)

Test set metrics urothelial cancer cells–segmented–EfficientNet-B0		
Accuracy (%)	F1-score (%)	MCC
Single best model	90.23 ± 0.44	90.06 ± 0.46
Ensemble	90.58 ± 0.43	90.24 ± 0.47

Together, these results confirm that the proposed label-free deep learning approach can accurately grade urothelial cancer cells based solely on quantitative phase-derived features, without the need for staining or manual extraction. This highlights its potential as an automated, objective, and scalable alternative to conventional tumor grading and possibly broader applications in treatment response assessment.

## Discussion

Recent advances have been made to push the limits of microscopy resolution, using principles of interference and holography to develop high-resolution imaging with phase holotomographic microscopy [36]. Recent works have begun to ask whether the high-resolution data provided by these novel methods could be coupled with ML, with machine-learning-influenced grading of pathology samples classified using quantitative phase imaging [37,38] for better image and morphological reconstruction [39], holotomography for morphological characterization [40], or even for the classification of cell types in blood samples or cancers of different organ origin [41].

Here, we present the first known instance of classification of chemotherapy-treated *vs.* untreated cells from the same cell population origin using coupled phase holotomographic microscopy with a CNN-type architecture. In addition, the generalizability of this approach is demonstrated by applying the model to grade a label-free, fixed, nonadherent cell line. We show that pairing label-free live-cell PHT with pretrained deep learning can classify A549 cells as PTX-treated or untreated with high accuracy. EfficientNet-B0 performed best, and an independent refractive-index shift in treated cells supports the presence of subtle biophysical changes that the model can exploit in characterization. Performance on bladder cancer cells indicates potential portability across multiple cell types.

Notably, the refractive image data indicate that, in the treated A549 cell population and the high-grade urothelial cancer cell population, approximately half of the cells are centered on a higher RI value. In the treated *vs.* untreated cell case, this could correspond with the fact that at the IC_50_ value, we expect half of the cells to respond to treatment by undergoing a cell death pathway. In the case of the high-grade urothelial cancer cells, this shift could correspond with increasing levels of heterogeneity, a known cause of metastasis, treatment resistance, and aggressiveness in later stages of cancer. However, notably, the ML models were able to classify cells at much higher than 50% accuracy (97% for the treated *vs.* untreated A549 and 91% for the high- *vs.* low-grade urothelial cancer cells), indicating that single measurements such as RI alone are insufficient for full characterization of these highly heterogeneous cell populations. Similarly, we can expect that these pipelines would also be useful (if provided with a training set for these features) for classifying different subtypes within these populations.

PHT alone is a valuable tool for imaging and characterizing subcellular features without labels, enabling high-resolution organelle-level visualization of live cells [42]. However, there are drawbacks to its implementation in quantitative analysis without the complementary use of ML. For instance, holographic reconstructions through thick material regularly overestimate the RI of all features inside the cell, and there are known difficulties in deconvolving sample thickness from RI, making quantitative RI labeling for the purpose of organelle classification very difficult [43], and features far away from the central plane of the reconstruction are often of lower image quality. In highly rounded or nonadherent cells, features such as organelles, the nucleus, and vesicles are obscured both by not being fixed on a substrate and by the amount of matter that the sample beam must pass through. However, even if these drawbacks impede visual analysis, signatures correlating RI with cell depth and clarity of organelles, computationally excluding human bias on the cell features most important for this classification, could surpass and, in fact, utilize the complexity of this information to determine various classifications of stage, treatment resistance, etc.

As this work represents a novel application of the combination of PHT with CNN-type ML, we acknowledge important constraints: *in vitro* data, a limited number of cell lines and one drug, class imbalance, and segmentation-related variability. Next steps will include expanding datasets across drugs, doses, and cancer types, as well as incorporating 3D models and artificial RI staining to increase data dimensionality. This pipeline should also be tested on a greater number of clinically relevant samples to assess robustness, including different drug types and concentrations, cell types, and staging *vs.* chemotherapy optimization cases. In the future, longitudinal studies evaluating the morphological cellular changes over time could also be studied.

## Conclusion

This work demonstrates the implementation of coupled ML with phase holotomographic microscopy for the label-free characterization of live adherent lung adenocarcinoma cells treated with chemotherapy as a proof-of-concept model for treatment optimization, as well as fixed nonadherent urothelial cancer cells as a proof-of-concept model for automatic tumor staging. The selection of these disparate cases and application of the same ML coupled to a holotomography imaging pipeline speaks to the potential generalizability of this approach. Rapid advances in the area of both ML and phase holotomographic microscopy can be expected to improve and advance this technique further. In the future, further refinement and testing of this approach could enable rapid, nondestructive readouts for drug testing, treatment optimization, and method development, reducing reliance on stains and complex sample preparation. Clinical samples can often be highly heterogeneous due to differences between laboratories, patient variability, and differences in imaging setups and protocols—challenges that will need to be considered in the long-term implementation of this approach. While this paper demonstrates an early proof of concept for a coupled holotomography/ML approach for cell classification, with appropriate validation, it may contribute to workflows for phenotypic screening and, in the longer term, decision support in personalized therapy research.

## Data Availability

The code is available on Github: github.com/leonorlosa/Thesis.
