# Supplementary material for: Leveraging Pretrained Neural Network Models for the Classification of Tumor Cells Analyzed by Label-Free Phase Holotomographic Microscopy
Source: Comput Struct Biotechnol J. 2026 Jun 9;35(1):0111. doi: 10.34133/csbj.0111 (PMC13247312; doi:10.34133/csbj.0111)
Supplement: Supplementary 1 — Figs. S1 to S6 [file csbj.0111.f1.docx]

Supplemental information

Title

Leveraging Pre-trained Neural Network Models for the Classification of Tumor Cells Analyzed by Label-free Phase Holotomographic Microscopy

**Authors**

Leonor V. C. Losa^#1^, Temple A. Douglas^#1^, Lia Santos^1^, Raquel Monteiro^2,3^, Isabel Calejo^2,3^, Raphaël F. Canadas^2,3^, Jana B. Nieder^1,*^

^1^ INL – International Iberian Nanotechnology Laboratory, Nieder Group on Ultrafast Bio- and Nanophotonics, Av. Mestre Jose Veiga s/n, 4719-330 Braga, Portugal

^2^ Department of Biomedicine, Faculty of Medicine, University of Porto, 4200-450 Porto, Portugal

^3^ RISE-Health, Faculty of Medicine, University of Porto, 4200-450 Porto, Portugal

^#^ these authors contributed equally to the work

^*^corresponding author: [jana.nieder@inl.int](mailto:jana.nieder@inl.int), [+351 253 140 112](https://www.google.com/search?q=inl+phone+number+braga+pt&sca_esv=47301926bc5cebf6&sxsrf=AE3TifOMoKxgOJJP89UuO5KiDaUWmvCNUA%3A1767372503255&ei=1_ZXaemnD7yy5NoPkaim6Ak&ved=0ahUKEwip7_fRp-2RAxU8GVkFHRGUCZ0Q4dUDCBE&uact=5&oq=inl+phone+number+braga+pt&gs_lp=Egxnd3Mtd2l6LXNlcnAiGWlubCBwaG9uZSBudW1iZXIgYnJhZ2EgcHQyBRAhGKABMgUQIRigATIFECEYoAEyBRAhGKABMgUQIRigATIFECEYqwIyBRAhGJ8FSKsLUH1Y2gpwAXgBkAEAmAF_oAGVB6oBAzAuOLgBA8gBAPgBAZgCCaAClAjCAgoQABhHGNYEGLADwgIGEAAYFhgewgILEAAYgAQYigUYhgPCAgUQABjvBcICCBAAGBYYHhgKmAMAiAYBkAYIkgcDMS44oAf_JrIHAzAuOLgHjQjCBwUyLTcuMsgHP4AIAQ&sclient=gws-wiz-serp)

*Dose response curve for PTX in A549 cells*

During determination of the IC50, the data were fitted to an [Inhibitor] vs. response - variable slope (four-parameter) model as shown in Figure S1.

Figure S1: Dose-curve response of PTX in A549 cells.

*Batch-removed test run of algorithm*

In order to analyze whether batch effects were contributing to the accuracy of the algorithm, we performed an additional analysis in which all of the images that were processed on a single day were removed from the initial training/validation dataset, to exclude some experimental batches completely from the training and validation pipeline. The excluded dataset was then used as a test set.

The confusion matrices for the test dataset across three different runs are shown below (Figure S2-S4):


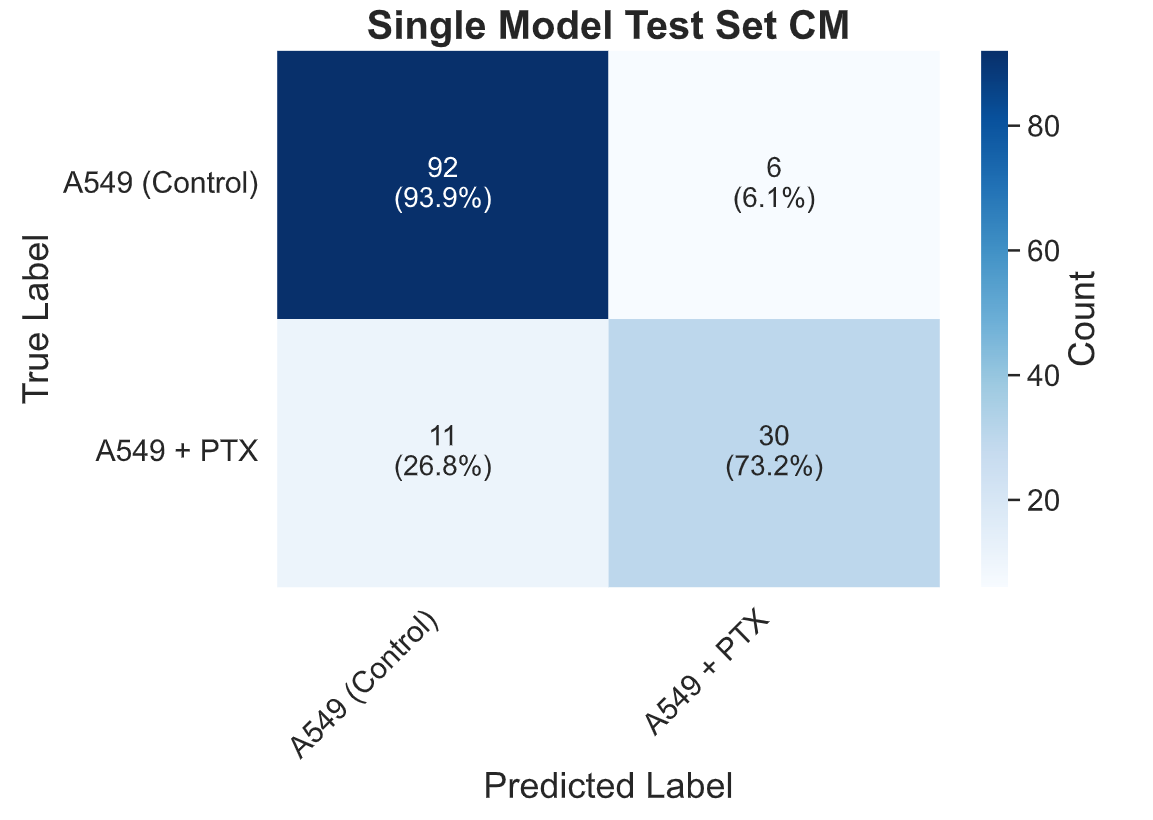

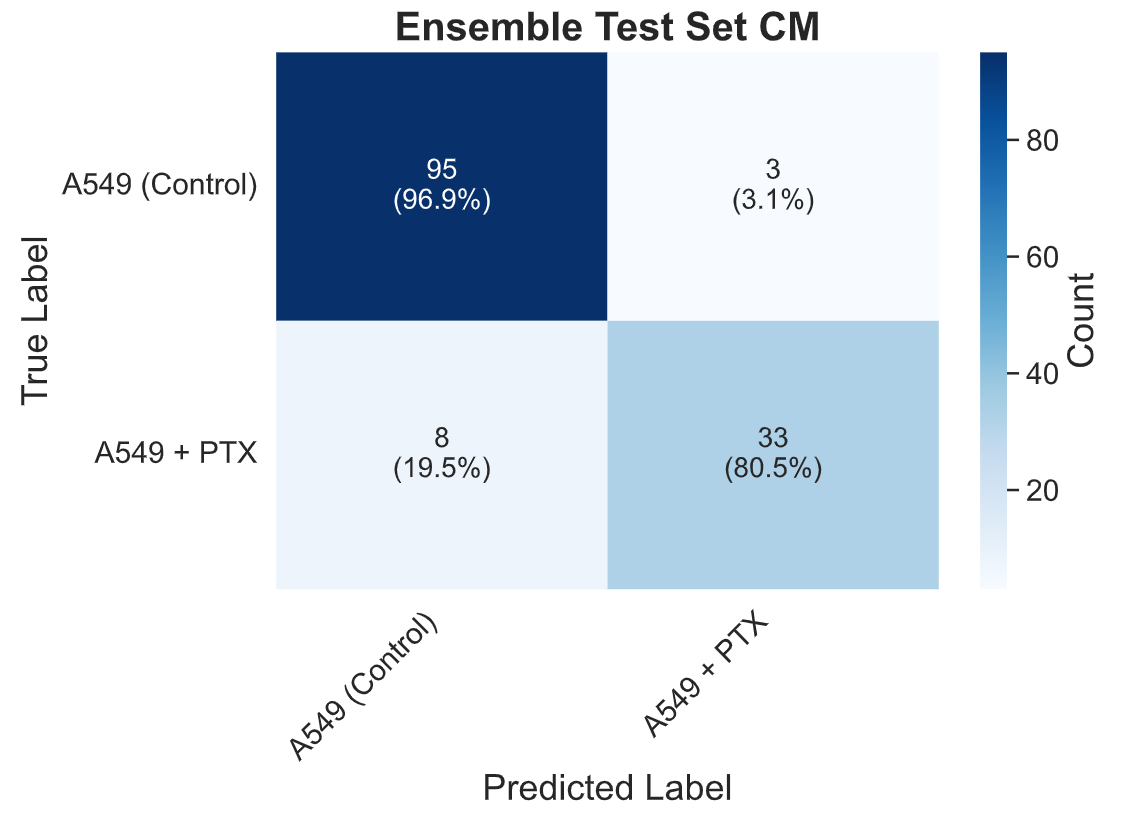


Figure S2: Single model and ensemble results for test set, first run.


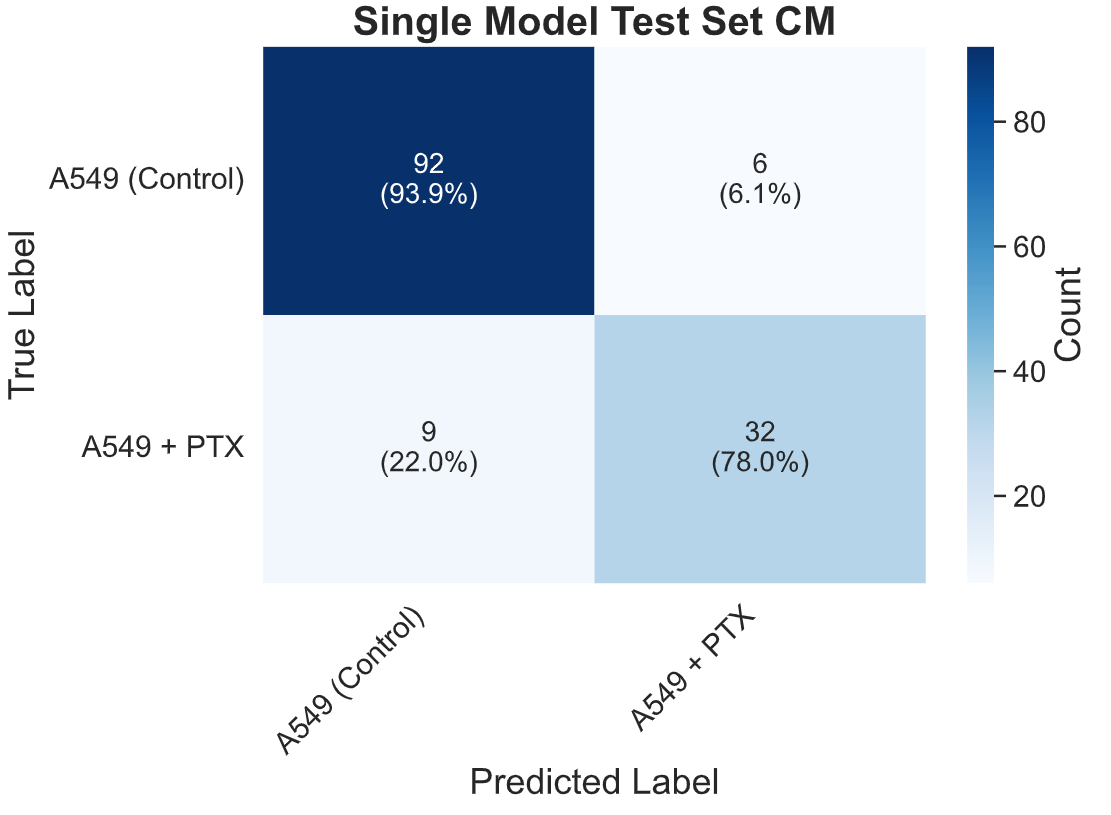

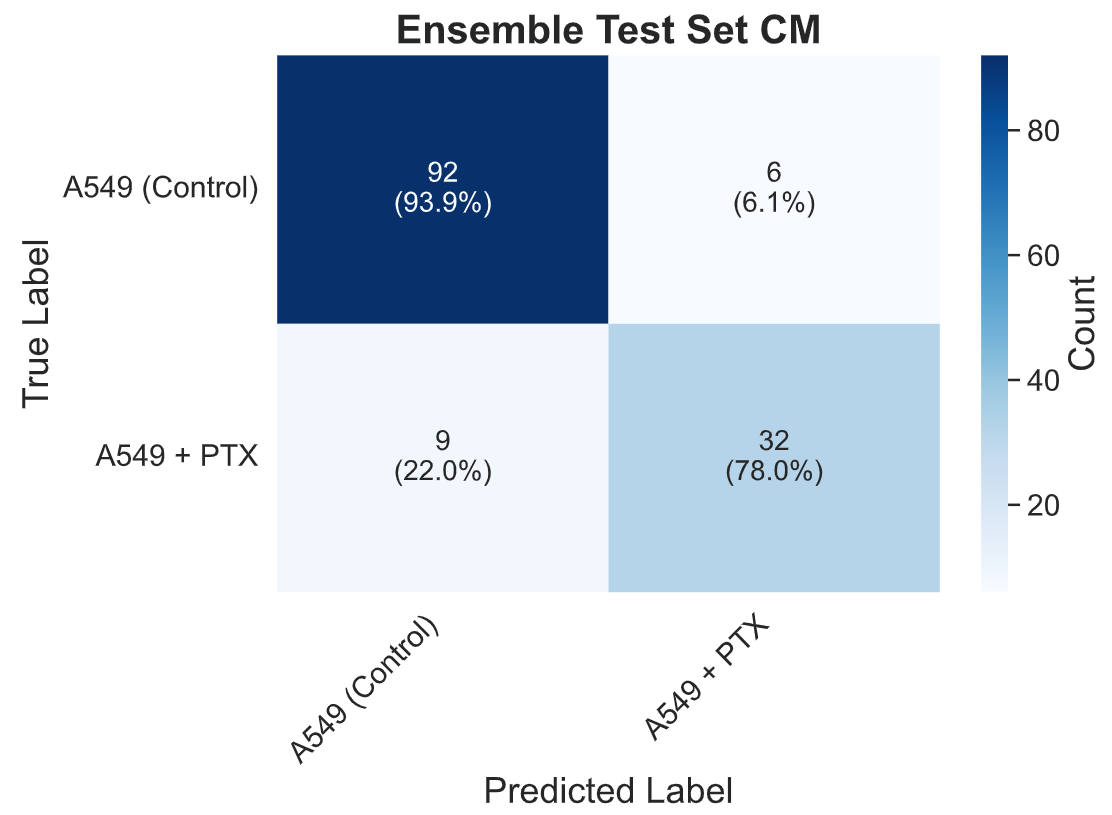


Figure S3: Single model and ensemble results for test set, second run.


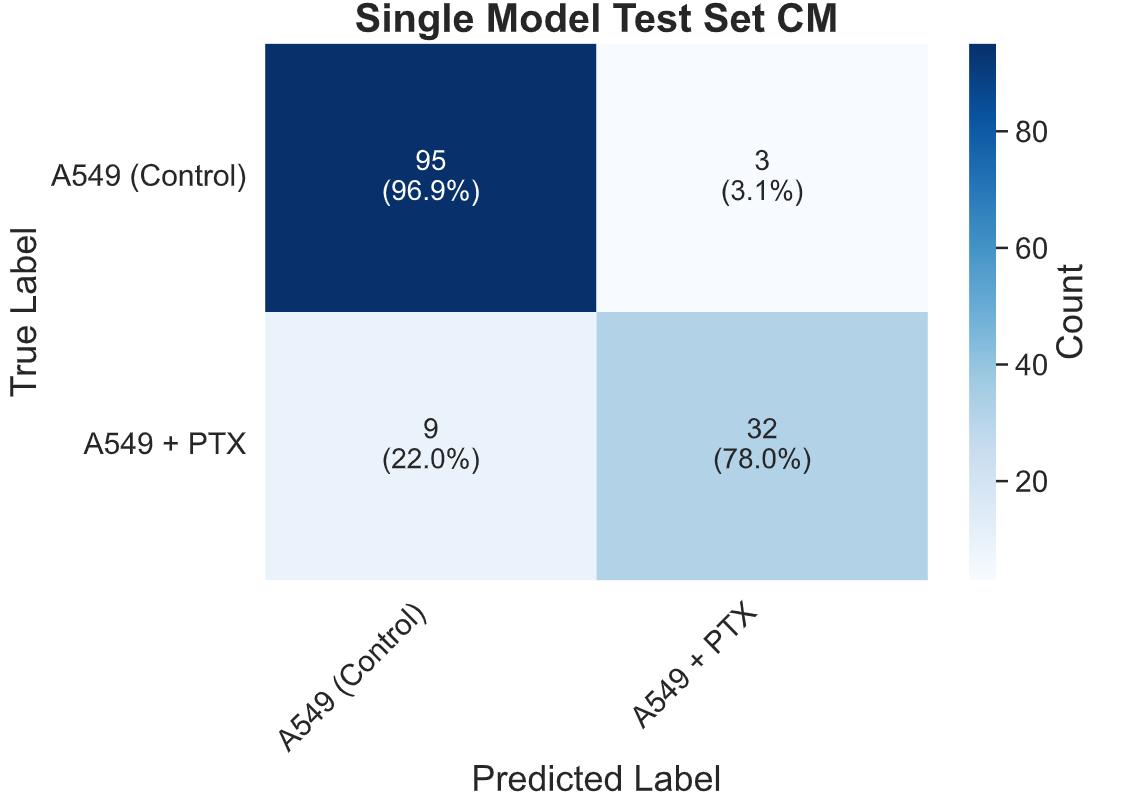

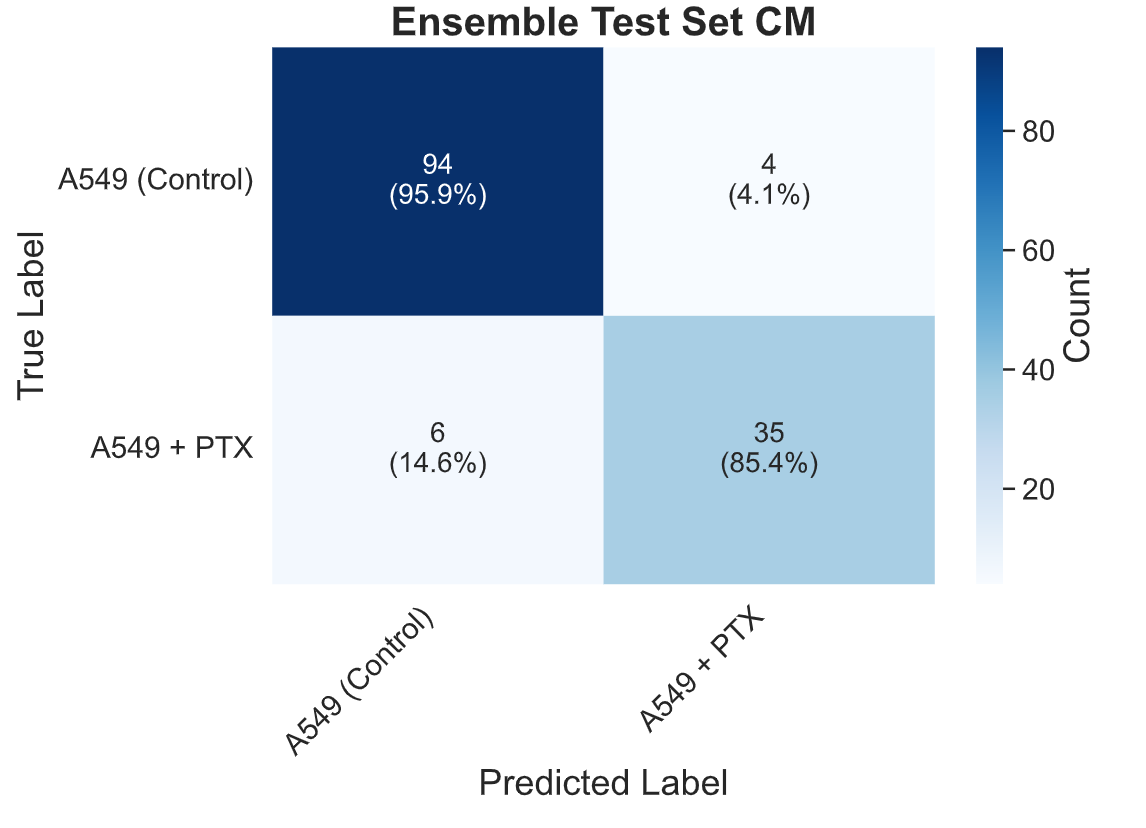


Figure S4: Single model and ensemble results for test set, third run.

The aggregating the results from these runs are shown in Figure S5 and S6:


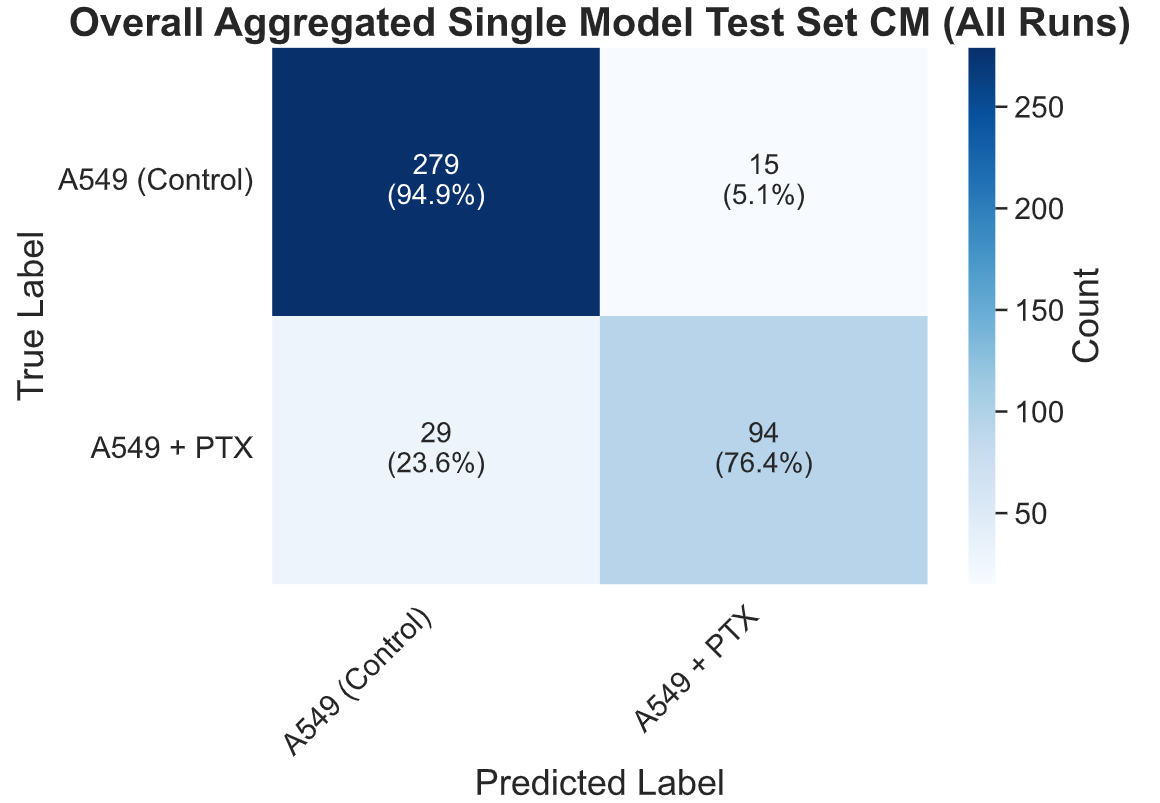

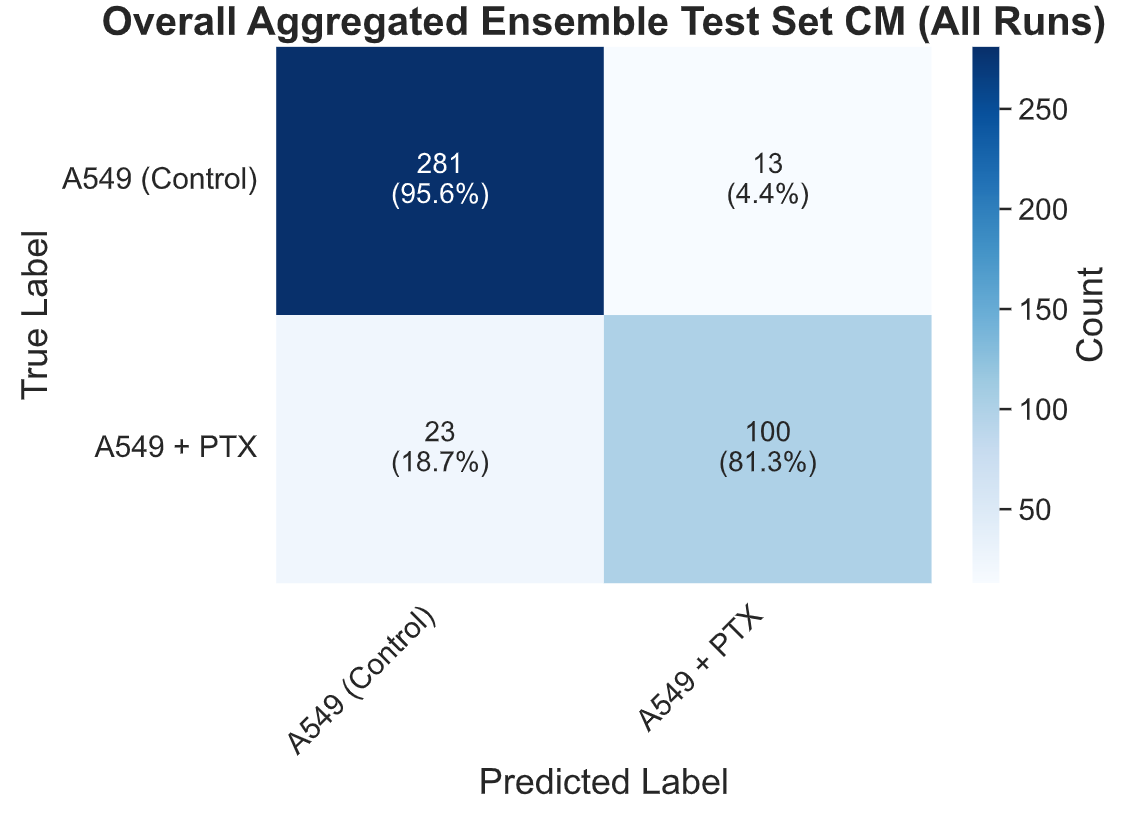


Figure S5: Aggregated results from test set run.


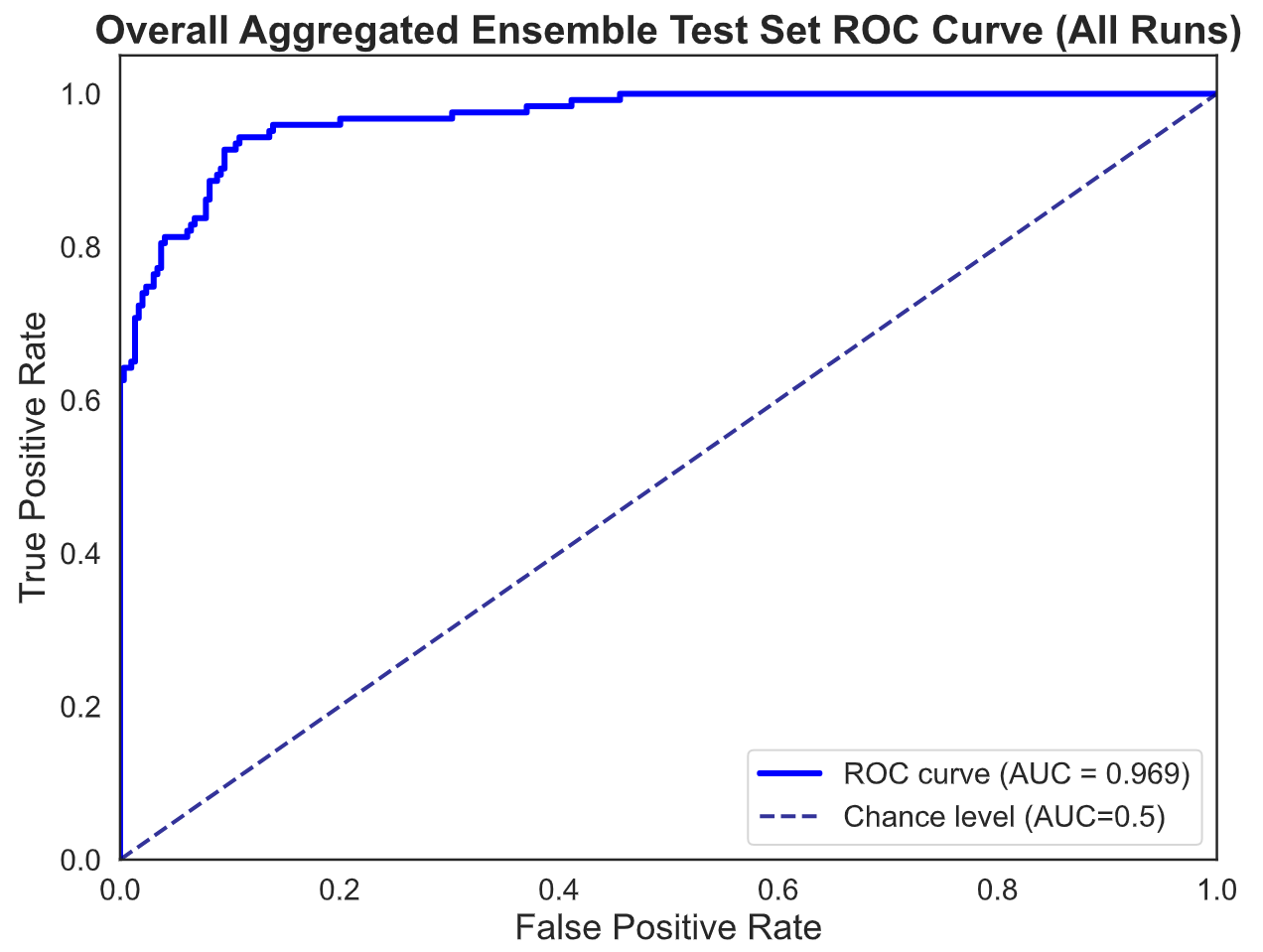


Figure S6: ROC curve for test set.

The precision and recall of the A549 population in the aggregated ensemble results was 0.92 and 0.96, respectively. In the A549-PTX population these values were 0.88 and 0.81. Averaging these recall values provides a balanced accuracy of 88%.
